# Author Correction: Timing and trajectory of *BCR::ABL1*-driven chronic myeloid leukaemia

**DOI:** 10.1038/s41586-026-11253-5

**Published:** 2026-10-12

**Authors:** Aleksandra E. Kamizela, Daniel Leongamornlert, Nicholas Williams, Xin Wang, Kudzai Nyamondo, Kevin Dawson, Michael Spencer Chapman, Jing Guo, Joe Lee, Karim Mane, Kate Milne, Anthony R. Green, Timothy Chevassut, Peter J. Campbell, Patrick T. Ellinor, Brian J. P. Huntly, E. Joanna Baxter, Jyoti Nangalia

**Affiliations:** 1 Wellcome Sanger Institute, Hinxton, UK; 2 Cambridge Stem Cell Institute and Department of Haematology, University of Cambridge, Cambridge, UK; 3 Cambridge University Hospitals NHS Trust, Cambridge, UK; 4 Cardiovascular Disease Initiative, Broad Institute of MIT and Harvard, Cambridge, MA USA; 5 Centre for Haemato-Oncology, Barts Cancer Institute, Queen Mary University of London, London, UK; 6 Department of Clinical and Experimental Medicine, Brighton and Sussex Medical School, Brighton, UK; 7 Cardiovascular Research Center, Massachusetts General Hospital, Boston, MA USA

**Keywords:** Cancer genetics, Haematological cancer, Phylogenomics, Evolutionary genetics

Correction to: *Nature* https://doi.org/10.1038/s41586-025-08817-2 Published online 9 April 2025

In the version of the article initially published, in the Author contributions section, the text “T.C. provided mentorship to A.E.K” should have read “T.C. contributed supervision, scientific advice and data review”. This correction has been made to the HTML and PDF versions of the article.

