# Supplementary material for: Timing and trajectory of BCR::ABL1-driven chronic myeloid leukaemia
Source: Nature. 2025 Apr 9;640(8060):982–90. doi: 10.1038/s41586-025-08817-2 (PMC12018454; doi:10.1038/s41586-025-08817-2)
Supplement: Supplementary file 1 — Supplementary Notes 1–4. [file 41586_2025_8817_MOESM1_ESM.pdf]

nature portfolio

https://doi.org/10.1038/s41586-025-08817-2

Supplementary information

Timing and trajectory of *BCR::ABL1*-driven chronic myeloid leukaemia

In the format provided by the authors and unedited

SUPPLEMENTARY NOTE 1: SNV mixed model analysis

To model the effect of *BCR::ABL1* fusion status on single nucleotide variant (SNV) burden (nsub_adj), we built the model in a stepwise fashion: (i) Confirm a *BCR::ABL1* fusion status effect as a fixed effect only, (ii) Test the addition of explanatory variables to the random effect configuration. Linear mixed models implemented in the R package "lme4" were used due to the repeated measures at the patient-level in the data set. Default "lme4" parameters were used and the model was estimated with maximum likelihood (REML=FALSE). Where models did not converge, lme4::allFit() was used to refit the model to all available optimisers ("lme4", "optimx", and "dfoptim" R packages), and the best optimiser was selected from non-singular and converged refits with the highest negative log-likelihood. Non-singular and converged models were considered for model selection using the Bayesian information criterion (BIC). As PD57332 did not have any wild-type colonies which would have biased SNV burden estimates, we removed this patient from the analysis, leaving 799 samples across 8 patients. Age (age_at_sample_exact) was defined as the count of completed years from birth at sampling and sample mutant status (*BCR_ABL1*) was defined as *BCR::ABL1* positive (Mt; n=330) or negative (Wt; n=469).

Using the subset dataset (n=799), we compared the inclusion of *BCR::ABL1* fusion status as a fixed effect (nsub_adj ~ age_at_sample_exact + *BCR_ABL1* + (1 | Patient)) to a null model "nsub_adj ~ age_at_sample_exact + (1 | Patient)". Adding *BCR::ABL1* fusion status (Model 1) significantly improved the model fit to the data (BIC=8762.57) over the null model (BIC=8907.52), confirming a *BCR::ABL1* fusion status effect. To improve the model, we next tested whether "Patient" has an effect on the slope and intercept in 2 models;

- **Model 2 - Age at sampling**
  nsub_adj ~ age_at_sample_exact + BCR_ABL1 + (1 + age_at_sample_exact | Patient)
- **Model 3 - *BCR::ABL1* fusion status**
  nsub_adj ~ age_at_sample_exact + BCR_ABL1 + (1 + BCR_ABL1 | Patient)

Both models were significant improvements over the baseline model's fit to the data (Model 1; BIC=8762.57), with Model 3 (BIC=8645.52) identified as the better model over Model 2 (BIC=8722.99). Removal of the random effect intercept did not improve the model further and the final model (refitted with REML) used in Figure 3a was as follows: $nsub\_adj \sim age\_at\_sample\_exact + BCR\_ABL1 + (1 + BCR\_ABL1 \,|\, Patient)$

Bootstrapped fixed effects CIs

Restricted to first 1000/3000 bootstrapped models

	SNV burden		
Predictors	*Estimate s*	*CI*	*p*
(Intercept)	36.45	-73.80 – 146.69	5.166e-01
Age(years)	18.13	16.21 – 20.06	**1.320e-63**
BCR::ABL1 status: Mt	91.72	33.66 – 149.77	**1.996e-03**
**Random Effects**			
σ^2	2556.59		
$\tau_{00\ Patient}$	4148.21		
$\tau_{11\ Patient.BCR\_ABL1Mt}$	5777.57		
$\rho_{01\ Patient}$	0.00		
ICC	0.72		
$N_{Patient}$	8		
Observations	799		
Marginal R^2 / Conditional R^2	0.829 / 0.952		

term	estimate	lower	upper	type	level
(Intercept)	36.45	-77.73	152.83	perc	0.95

age_at_sample_exact	18.13	16.07	20.15	perc	0.95
*BCR_ABL1*Mt	91.72	30.58	150.39	perc	0.95

Restricted to 884 converged non-singular bootstrapped models

term	estimate	lower	upper	type	level
(Intercept)	36.45	-76.48	152.62	perc	0.95
age_at_sample_exact	18.13	16.09	20.19	perc	0.95
*BCR_ABL1*Mt	91.72	33.63	151.58	perc	0.95

SUPPLEMENTARY NOTE 2: Telomere mixed model analysis

Mitchell *et al*[1] showed that *telomerecat* telomere length estimates from Illumina NovaSeq sequenced samples were unusual with very high and zero length estimates compared to Illumina HiSeqX sequenced samples. We hypothesised that a likely contributing cause is the change from 4 colour (HiSeqX) to 2 colour (NovaSeq) sequencing. Therefore, NovaSeq sequencing outputs a "G" when there is a "G" base and when there is an error, causing misattribution of telomeric reads by *telomerecat*. Reads containing telomeric repeats (from *telomerecat* telbam) show a marked increase in "G" content after 75bp. Running *telomerecat* with -t 75 "trims" read *in silico* to the first 75bp when allocating read type results in a decrease in outlier estimates. *Telomerecat* estimation also demonstrated batch effects. *Telomerecat* estimates the mean telomere length using counts of telomeric reads (F1) and telomere boundary reads (F2_a). This makes it robust to ploidy with the assumption that F1 and F2_a reads are recalled at the same rate. We hypothesised that this assumption could be affected by (i) Lower read depth - causing telomere boundary reads (F2_a) to be stochastically under sampled, thereby, giving an overestimate of telomere length, and (ii) Sequencing library preparation - causing batch to batch variation in the ratio of F1/F2a reads. Therefore, we defined two candidate batch variables: (i) The library preparation cluster (library.cluster), that is the library preparation date clustered by adjacent days (n=22), and (ii) The unique sequence run ID (run_id.uniq) which is the concatenated flow cell IDs of for each sample (n=31). Indeed, using "run_id.uniq" accounts for both library preparation clusters and sequencing batches.

To identify an effective batch effect variable, we restricted the data to *BCR*::*ABL1*-negative samples (n=469) and used linear mixed models (implemented as previously described), to compare each candidate batch effect variable to a baseline model with "age at sample" as a fixed effect and "patient" as a random effect.

- **Model 0 - Baseline**
  Length ~ age_at_sample_exact + (1 | Patient)

- **Model 0b - library.cluster**
  Length ~ age_at_sample_exact + (1 | Patient) + (1 | library.cluster)

- **Model 0c - run_id.uniq**
  Length ~ age_at_sample_exact + (1 | Patient) + (1 | run_id.uniq)

Across all models, mean telomere length attrition rates estimates (per year) were compatible with Mitchell *et al*[1] showing that NovaSeq derived *telomerecat* mean telomere length can be used successfully to estimate telomere reads following correction for batch effects. Both candidate batch variables improve the baseline model's fit to the data, with " + (1 | run_id.uniq)" (Model 0c; BIC=7483.33) identified as the better variable over " + (1 | library.cluster)" (Model 0b; BIC=7507.51).

We next modelled the effect of *BCR::ABL1* fusion status on mean telomere length, and built the model in a stepwise fashion: (i) Confirm a *BCR::ABL1* fusion status effect as a fixed effect only, (ii) Test the addition of explanatory variables to the random effect configuration. Using the full dataset (n=834), we compared the inclusion of *BCR::ABL1* fusion status as a fixed effect to a null model "Length ~ age_at_sample_exact + (1 | Patient) + (1| run_id.uniq)". Adding *BCR::ABL1* fusion status as a fixed effect improves the model fit to the data (Model 1; BIC=13266.02) over the null model (Model 0c; BIC=13299.03), confirming a significant *BCR::ABL1* fusion status effect. To improve the model further, we tested if "Patient" has an effect on the slope and intercept in 2 models;

- **Model 2 - Age at sampling**
  Length ~ age_at_sample_exact + BCR_ABL1 + (1 + age_at_sample_exact | Patient) + (1 | run_id.uniq)
- **Model 3 - *BCR::ABL1* fusion status**
  Length ~ age_at_sample_exact + BCR_ABL1 + (1 + BCR_ABL1 | Patient)+ (1 | run_id.uniq)

Model 3 converged and was an improvement (BIC=13265.15) over the baseline model (Model 1; BIC=13266.02).

The final model used in Figure 3g was as follows: $Length \sim age\_at\_sample\_exact + BCR\_ABL1 + (1 + BCR\_ABL1 \mid Patient) + (1 \mid run\_id.uniq)$

	Mean telomere length(bp)		
Predictors	*Estimates*	*std. Error*	*Statistic*
(Intercept)	6179.88	444.18	13.91
Age(years)	-30.79	8.15	-3.78
BCR::ABL1 status: Mt	-556.90	225.06	-2.47
**Random Effects**			
σ^2	399685.76		
$\tau_{00\ run\_id.uniq}$	211135.35		
$\tau_{00\ Patient}$	24643.27		
$\tau_{11\ Patient.BCR\_ABL1Mt}$	311111.95		
$\rho_{01\ Patient}$	-0.33		
ICC	0.46		
$N_{Patient}$	9		
$N_{run\_id.uniq}$	31		
Observations	834		
Marginal R^2 / Conditional R^2	0.223 / 0.584		

Bootstrapped fixed effects CIs

Restricted to first 1000/3000 bootstrapped models

term	estimate	lower	upper	type	level
(Intercept)	6179.88	5207.41	7098.60	perc	0.95
age_at_sample_exact	-30.79	-47.83	-13.24	perc	0.95
*BCR_ABL1*Mt	-556.90	-988.32	-103.54	perc	0.95

Restricted to first 1000/1566 converged non-singular bootstrapped models

term	estimate	lower	upper	type	level
(Intercept)	6179.88	5216.22	7086.12	perc	0.95
age_at_sample_exact	-30.79	-48.16	-12.59	perc	0.95
*BCR_ABL1*Mt	-556.90	-993.16	-85.98	perc	0.95

SUPPLEMENTARY NOTE 3: Estimating *BCR::ABL1* growth rates

In the absence of longitudinal measurements of the *BCR::ABL1* clone VAF prior to diagnosis, we estimated the rate of growth of *BCR::ABL1* clones using the single cell-derived colony based phylogenetic trees and the pattern of coalescences of the *BCR::ABL1* mutant clade. To minimise the use of phylogenetic information that may be affected by therapy, we subset the trees to use only those single cell derived colonies that were grown from cells extracted at the earliest diagnostic or post-diagnostic timepoint. We have 3 donors where the earliest sampling time point is after diagnosis. One of these, PD57333, has no detectable clone. The other two are PD57334 and PD57335. To infer

the growth rates we use our previously published method , *Phylofit*[1,2], and corroborate the results using a suite of methods available in the R package "*cloneRate*"[3]. Both *Phylofit* and *cloneRate* estimate growth based on the phylogeny of the mutant clade. *Phylofit* and *cloneRate*'s "maxLikelihood" require the tree to be time-based ultrametric trees. We infer a time-based tree, including all available sampling time points, using our previously developed R package "*rtreefit*" (https://github.com/NickWilliamsSanger/rtreefit, further detailed in Williams *et al*[2]) under the assumption that the observed per branch mutations are Poisson distributed.

Timing *BCR::ABL1* clonal expansion

The *rtreefit* method allows for the transition between mutant and wild-type rates to occur a fixed fraction down the branch on which the mutation is acquired, which is the shared branch upstream of the *BCR::ABL1* positive clonal expansion (**Fig.2**, showing the upstream 'trunk' of the CML clade, and marked as Pre-CML lineage in **Fig.3b**). As shown in **Fig.3e**, the signature analysis suggests *BCR::ABL1* clonal expansion commences towards the very end of this branch given its mutation profile is indistinguishable from that of wild-type cells. We therefore configured *rtreefit* to set the acquisition as occurring at the very end of the branch (rtreefit::fit_clade parameter: xcross=0.99).

A post-hoc justification for the late positioning of the acquisition on this ancestral branch is also furnished by the resulting growth rate estimates. Simulations indicate that the expected time between acquisition and the MRCA of a large mutant clade is approximately exponentially distributed with a mean of $1/s$ (**Figure S1a**), indeed this is consistent with a known result for birth only processes[4]. This implies that the acquisition of *BCR::ABL1,* and the start of clonal expansion is likely to have occurred towards the end of the shared trunk. The resulting estimated gap between acquisition and the end of the branch is shown in **Figure S1b** and with **S1c** showing that our choice of xcross=0.99 is sensible. Given the more rapid acquisition of somatic mutations within the mutant clade, clade specific rates are inferred by *rtreefit* for clonal expansions.

This is strong evidence that the acquisition of *BCR::ABL1* (or the commencement of *BCR::ABL1* clonal expansion) is occurring at the very end of the shared branch upstream of the clonal expansion, particularly in patients where the subsequent clonal expansion rate is very high. In those patients that have intermediate growth rates, *BCR::ABL1* acquisition (or the commencement of *BCR::ABL1* clonal expansion) is still likely within ~1 year of the end of this shared branch.

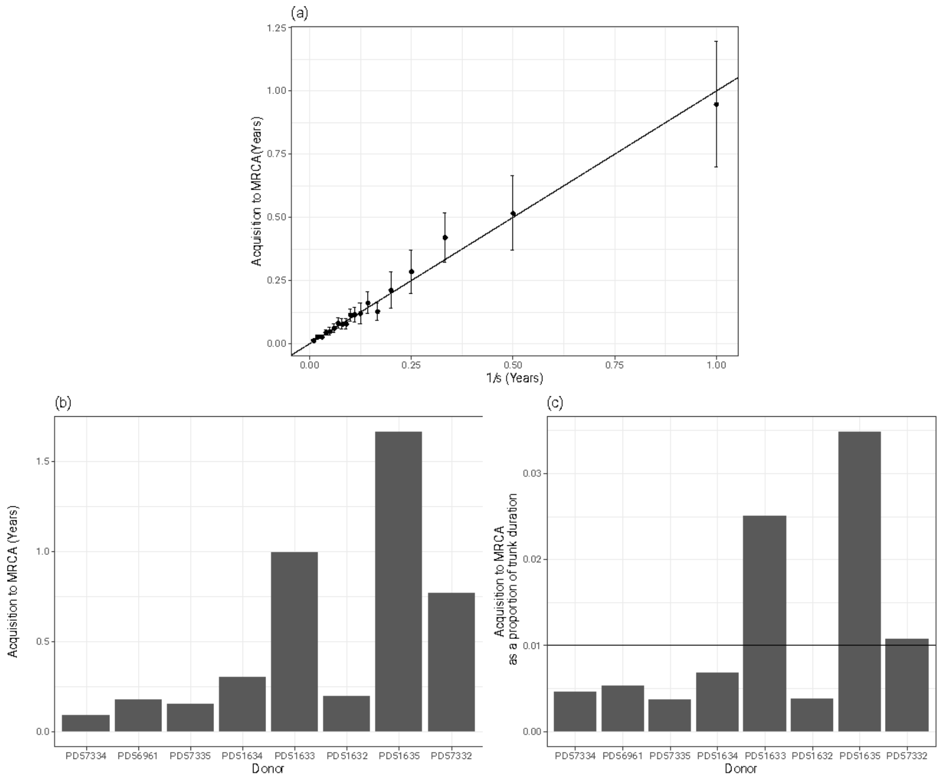

Figure S1: a) *Time between driver acquisition (or commencement of BCR::ABL1 clonal expansion) and timing of the most recent common ancestor ("gap") of a sampled clade with 100 cells. The error bars show the 95% confidence intervals for the mean gap based on the observed gap in 50 simulations for each value of the growth rate s.* ***b)***

Estimated time between BCR::ABL1 acquisition (or commencement of BCR::ABL1 clonal expansion) and the MRCA, based on the Phylofit inferred growth rate. ***c)*** *Estimated expected time between BCR::ABL1 acquisition (or commencement of BCR::ABL1 clonal expansion) and MRCA as a proportion of mutant trunk length. The gaps range from 0.004 to 0.035 indicating the acquisition is expected to have taken place very much towards the end of the branch.*

Comparison between *cloneRate* and *Phylofit* growth estimates

Johnson *et al*[3] developed a suite of methods for estimating birth-death models with a constant net growth rate. The estimates and associated confidence intervals are asymptotically correct in the limit of a large n (the number of samples in the sampled mutant clade), whilst n remains much smaller than the underlying population. In practice the authors recommend only using the approaches when $n > 10$. To validate our results using *phylofit,* we compared them to the results for two of their methods **(Figure S2)**:

- "birthDeathMCMC": This uses the exact likelihood equation of the constant parameter birth-death process in a Bayesian model to estimate the birth and death rates. As with all the cloneRate methods the estimation essentially assumes a pure exponential growth process.
- "maxLikelihood": This performs a maximum likelihood fit for the observed coalescence times under a model where the coalescence times are independent and identically distributed with an underlying logistic distribution. The growth rate is essentially estimated as being inversely related to the spread of the coalescence timings. We fit the model using our *rtreefit* time-based trees inferred using clade specific rates..
- "phylofit": This fits a growth rate based on a three-parameter logistic growth curve. We fit the model using the *rtreefit* time-based trees.

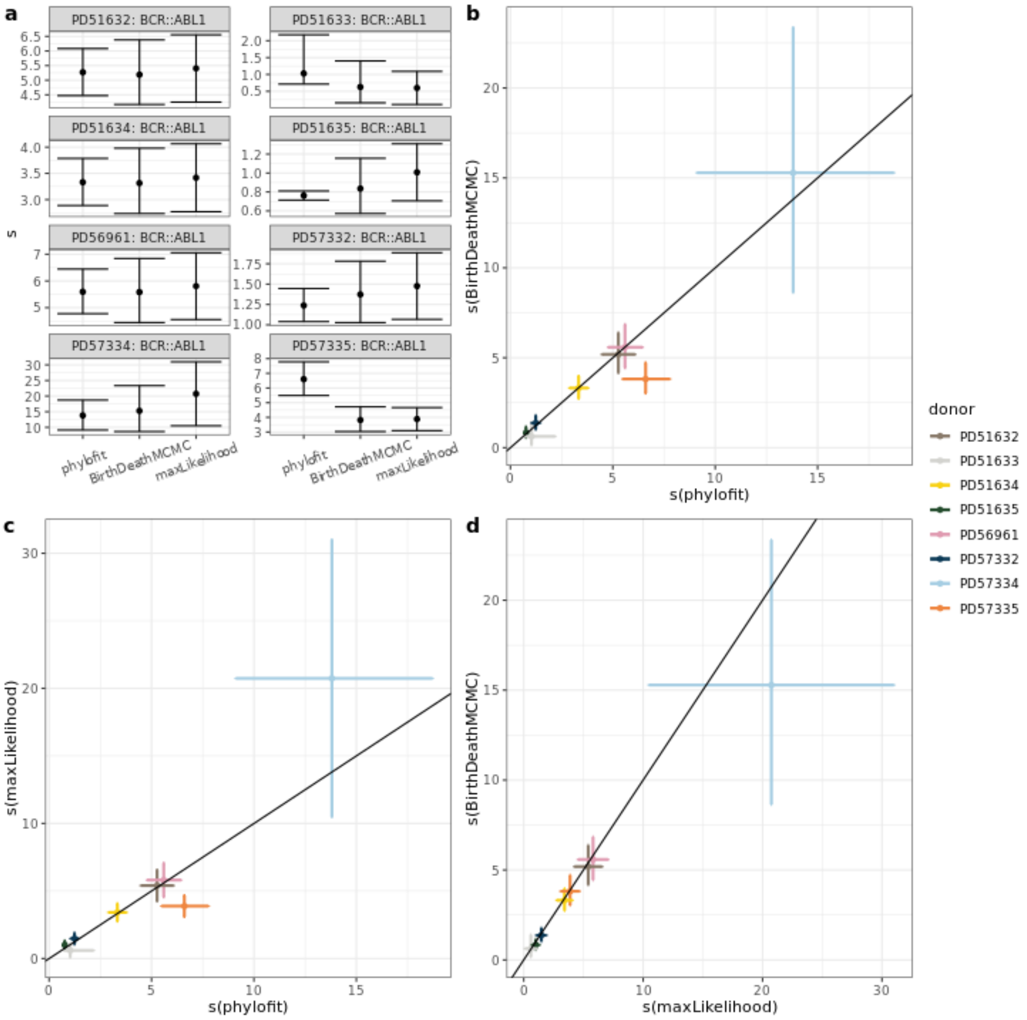

Figure S2 Alternative Growth Rate Estimates for the BCR::ABL1 expansions. *The error bars are 95% equal tailed credibility intervals for phylofit and birthDeathMCMC and 95% Confidence Intervals for the maxLikelihood method. The results are broadly comparable across the methods, except for PD51635 which has an atypical, staggered pattern of clonal diversification indicative perhaps of non-constant growth, and PD57335 which has some late coalescences which are perhaps due to TKI induced clonal reduction prior to the first sampling point.*

Removing late coalescences from PD57335 restores comparability between *Phylofit* and *cloneRate*

PD57335 is an outlier with respect to the *cloneRate* results (**Figure S2a-c**). The *cloneRate* estimates are strongly affected by the presence of late coalescences. Dropping 3 selected samples to remove the 3 late coalescences in this phylogenetic tree has very little effect on *phylofit* (s=6.602 goes to s=6.596), but markedly increases the *cloneRate* estimates (*birthDeathMCMC*:s=3.82 goes to s=6.10; *maxLikelihood* s=3.88 goes to 6.26) to approximately match the estimate of *phylofit.* PD57335 is also sampled a year after diagnosis, and growth rates inferred using this time point are relatively consistent across methods (s=7.78,7.29 and 7.39 for *phylofit, birthDeathMCMC* and *maxLikelihood* respectively) and are also quite close to the estimates made excluding the late coalescences in the first time point. Under these circumstances it seems likely that *Phylofit* better captures the early growth rate applicable before treatment.

Benchmarking of *Phylofit* and *cloneRate* for large growth rates

Neither *phylofit* nor *cloneRate* methods have previously been benchmarked for their accuracy in inferring the very large growth rates of the magnitude that are evident in some of the *BCR::ABL1* positive expansions. In Johnson *et al*[3] they benchmarked *cloneRate*, *birthDeathMCMC* and *phylofit* and concluded that *cloneRate* was slightly less accurate for small n but that both *cloneRate* and *birthDeathMCMC* are more accurate for large n and that *Phylofit*'s CI's tended to be too narrow. In benchmarking the methods for large growth rates in the presence of limited saturation of the population size we made similar findings. However, if we follow expansions into an extended period of mutant population saturation, which is the scenario *phylofit* was designed to accommodate, we find that the relative inference performance of *phylofit* improves. The simulation of clonal expansions are generated using *rsimpop* as described in Williams *et al*[1], where the birth rate of mutant cells differs from the birth rate of wild-type cells, here set to 1 symmetric division per year, by "s" but both cell types have the same death rate which is set as the weighted average of the birth rates so that the expected overall population size remains the same. In the early stages of the expansion when the size of the mutant clone is small, the death rate is approximately the birth rate of the wild-type and so the growth rate of the mutant clone is "s". The simulations were set to terminate when there is a 99.99% probability that a mutant population evolving under a constant birth death process with the specified "s" has reached a population of 100,000.

We benchmarked the three methods using 1000 simulated trees for each of 6 growth rates regimes (S=1, 10, 100, 1000, 10000, 100000) and 7 sampled clade sizes (4, 11, 32, 58, 75 and 91) corresponding to mutant clade sizes and the range of inferred growth rates in our CML cohort. For comparability, *maxLikelihood* estimates were capped at the upper end of the prior range used for the Bayesian methods ($s_{max} = 10$ (equivalent to S=22025) for S=1 and 10, and $s_{max} = 30$ (equivalent to S=1.07×10^{13}) for S>=100). The benchmarking indicates that all methods are somewhat inaccurate for low n (in our case 4 and 11), and that *birthDeathMCMC* and *Phylofit* are similarly accurate for moderate *n*, however for large *n*, *cloneRate* methods appear to be less accurate **(Figure S3).** The source of the emerging difference in accuracy for large n is evident in **Figure S4** where *cloneRate* methods tend to underestimate the growth in this limit - presumably because of the presence of late coalescences induced by the saturation of the growth of the mutant population is not compatible with very rapid pure exponential growth.

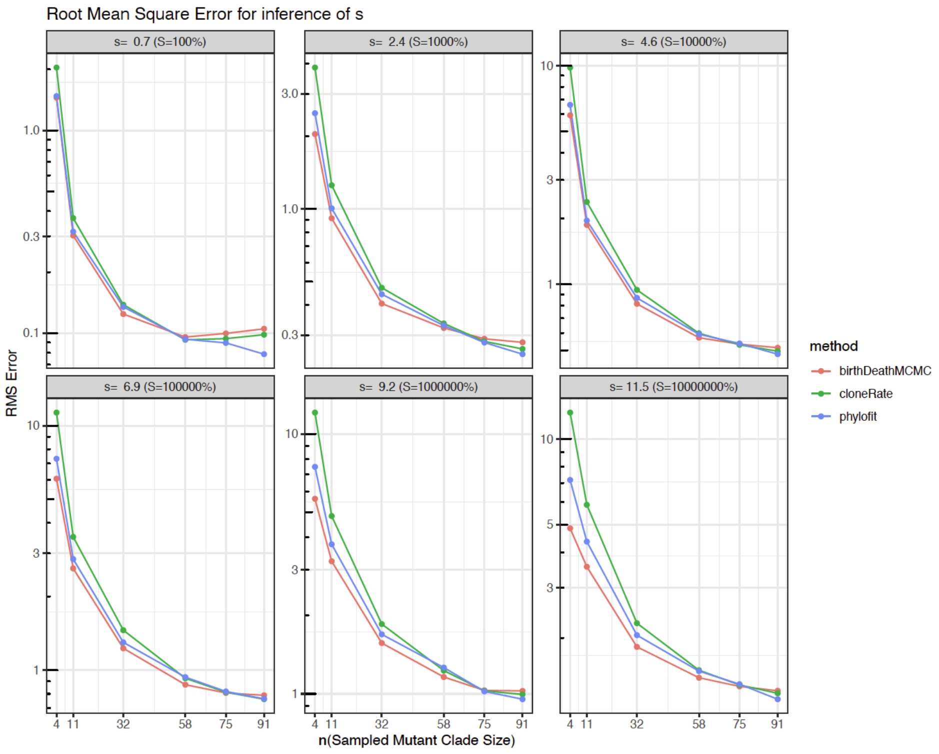

Figure S3 Root Mean Square Error in Growth Rate Estimation. *This plot shows that the cloneRate methods and Phylofit are similarly accurate in their central estimate of growth. For very low values of n (4 and 11) none of the methods performs well, but birthDeathMCMC and Phylofit are more accurate.*

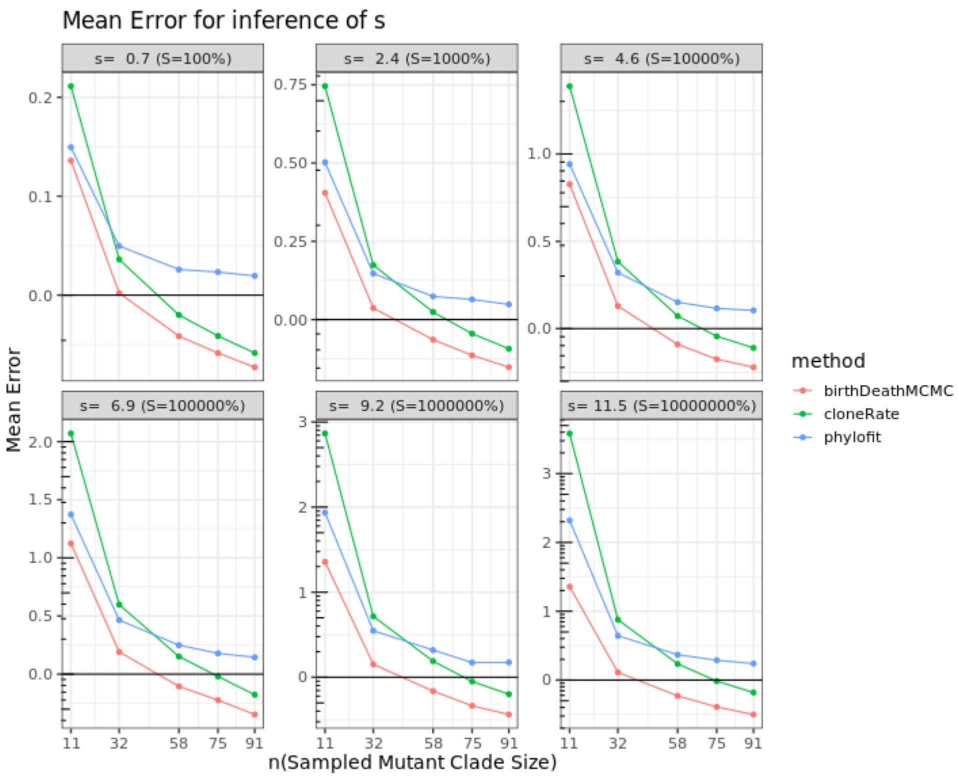

Figure S4 Mean Error in Growth Rate Estimation. *This shows that for saturated simulated trajectories the cloneRate methods tend to underestimate "s" whilst Phylofit overestimates "s" but tends towards the correct estimate for large n.*

Simulated history of a hypothetical CML case

Here, we use a case study of a hypothetical CML case to examine the robustness of the estimation methods of the initial phase growth rate based on trees sampled from a mutant population at various longitudinal timepoints from a mutant population with a reasonably realistic mutant cell population size trajectory as illustrated in **Figure S5a. Figures S5b-d** show example sampled trees from the mutant cell population at highlighted timepoints in **Figure S5a.** The figures illustrate that in the presence of unconstrained growth, e.g. between time point **a** and **b,** all methods give reasonably good inferences. Following periods of saturation **(Figure 5c)** and population contraction **(Figure 5d),** *phylofit* gives estimates that are reasonably accurate and consistent across trees sampled at the same time point, whereas the other methods give highly variable estimates depending on whether late coalescences are captured.

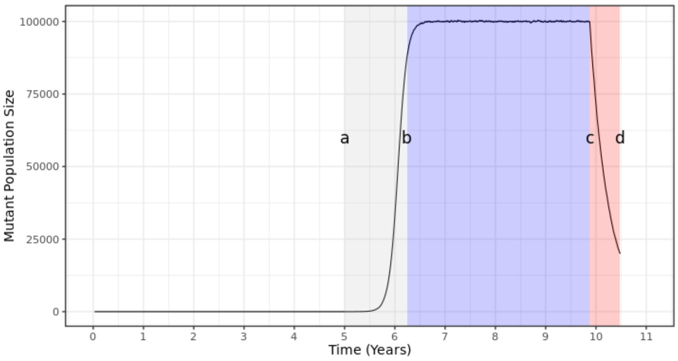

Figure S5a. *The driver is acquired at a) 5 years and introduced into an HSC population of constant size where the same death rate grows rapidly (s=11) until the end of the period. At b) trees are sampled, and then the clone carries on growing and then remains at the HSC carrying capacity (here 100,000 cells) for approximately 3 years. At c) trees are sampled again, and then the donor undergoes TKI reducing the mutant population by apoptosis (increased death rate). Finally as d) trees are sampled again.*

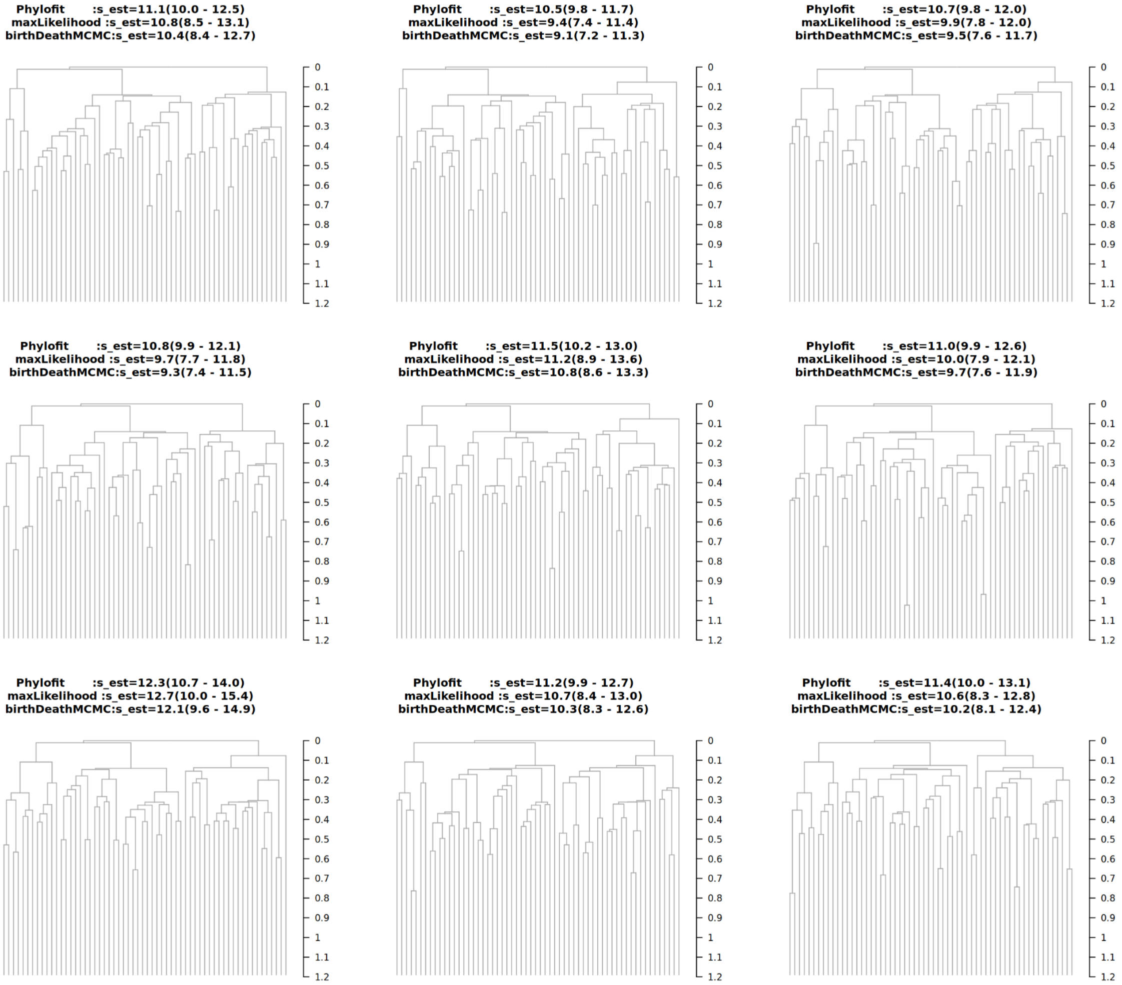

Figure S5b. *Trees sampled immediately at the end of the period of rapid growth at time point b. The figure shows all methods adequately recover the true growth rate (s=11).*

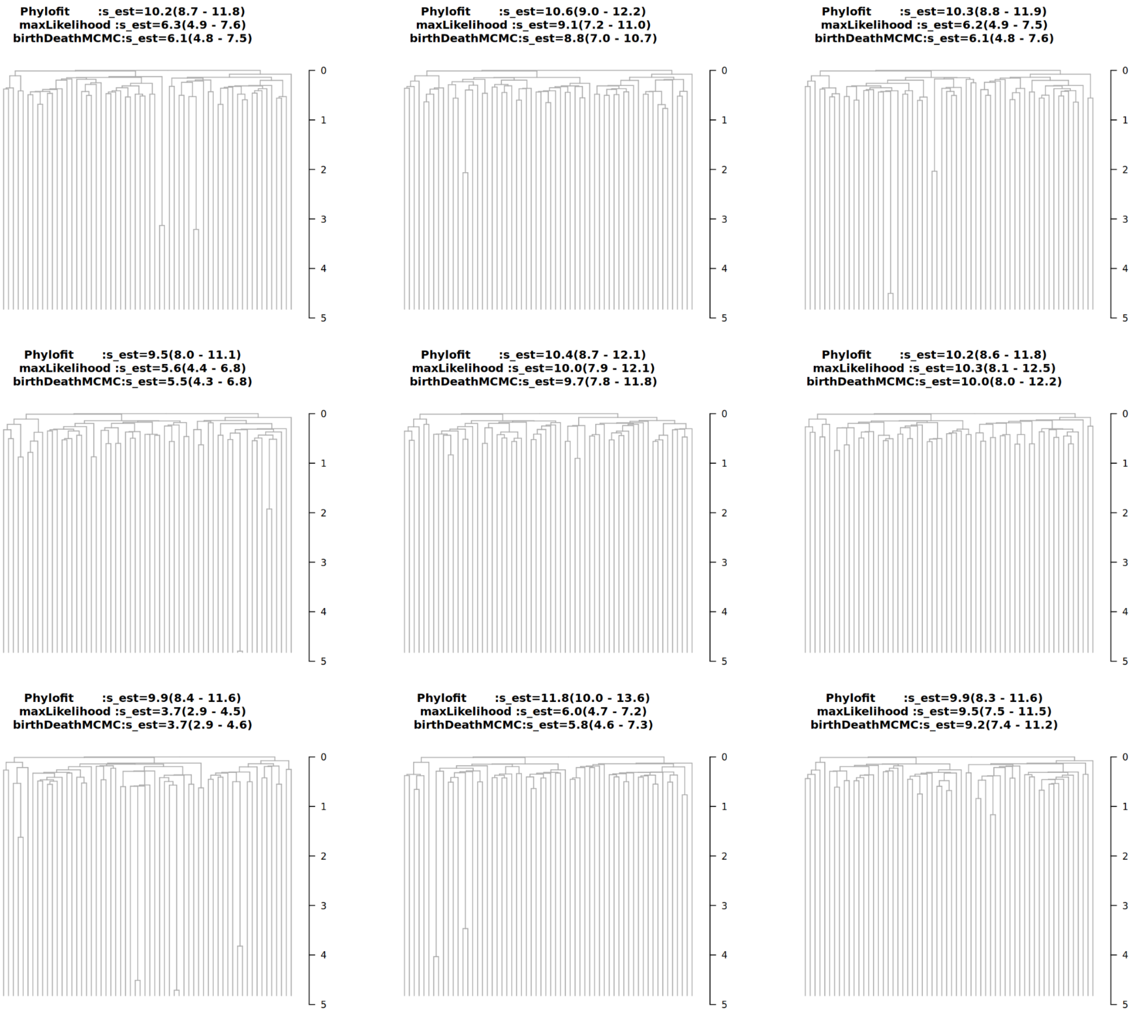

Figure S5c. *Trees are sampled at time c at the end of a prolonged period where the mutant population is at carrying capacity. For this population Phylofit generally adequately recovers the true growth rate. However, the cloneRate methods tend to markedly underestimate the growth rate in the five cases where late coalescences have been captured.*

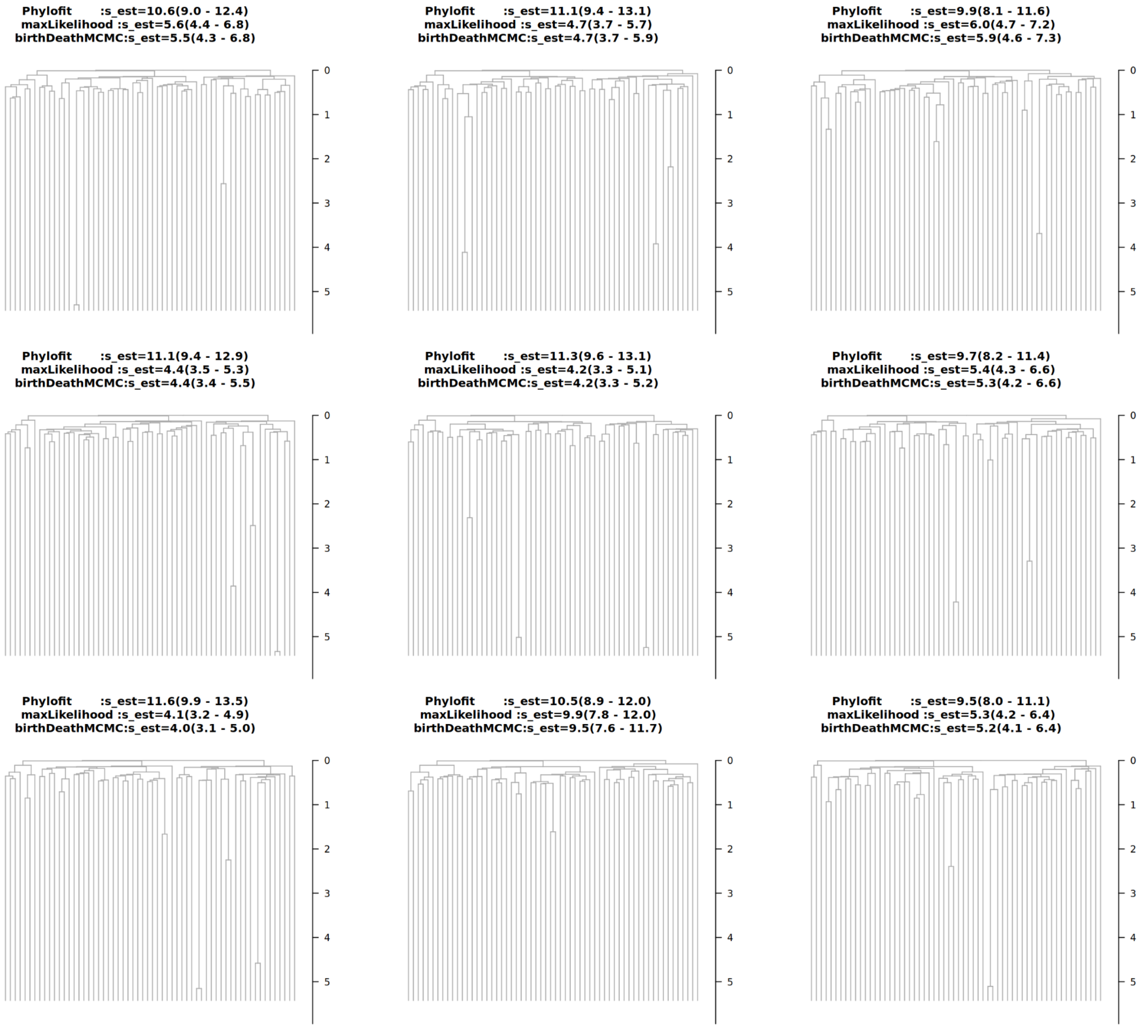

Figure S5d. *Trees sampled at time d at the point where the TKI has reduced the mutant clone by about 80%. For this population Phylofit still generally adequately recovers the true growth rate. However, the cloneRate methods markedly underestimate the growth rate in the eight cases where late coalescences have been captured.*

SUPPLEMENTARY NOTE 4: Relationship between latency and growth rate

To motivate the functional form of the relationship between growth rate of the *BCR::ABL1* clone and latency, defined as the duration from the start of the clonal expansion to clinical diagnosis, we consider a simplified model. Starting from a single stem cell we assume that it grows deterministically at an annual rate of S and that CML is diagnosed once there are a threshold number of mutant stem cells N^*. Thus, we have the following relationship between latency and s:

$$e^{s \times Latency_i} = N^*$$

This also gives the expected population size under a birth-death model with net growth rate s. If we use the above equation with our inferred values for latency and growth rate, we derive somewhat unrealistically high values for N^* **(Figure S6).** The highest estimates for N* are > 10^{19} for PD57334, the youngest CML patient in the cohort, and > 10^{12} for PD57335. These mathematical estimates are physiologically implausible, but the high mutant stem cell burden suggested by the growth rates is in keeping with the severe clinical presentation of these two individuals who suffered retinal haemorrhages secondary to circulating tumour load. It is likely that the growth rates slows down with time such that such N* numbers of stem cells are not reached.

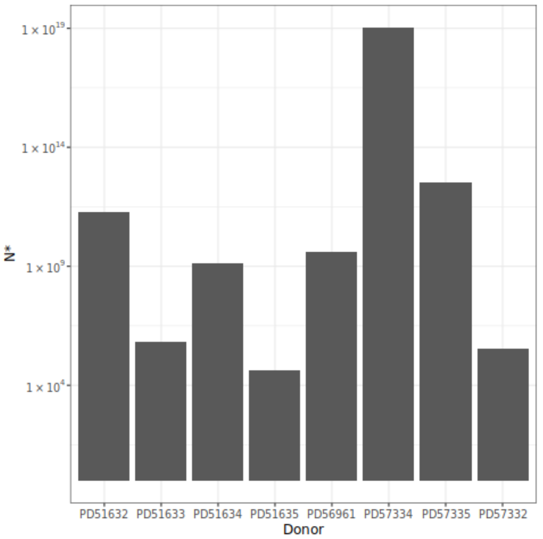

Figure S6 *Inferred mutant stem cell number at diagnosis*

Rearranging $e^{s \times Latency_i} = N^*$ we get :

$$log(s_i) = log(log(N^*)) - log(Latency_i)$$

The model fit is as follows: log(s) ~ log(Latency)

	Est.	S.E.	t.val.	p
(Intercept)	4.478357	0.2182320	20.52109	8.709e-07
log(Latency)	-1.786532	0.1120933	-15.93790	3.873e-06

Adjusted $R^2 = 0.97$

The model is an excellent fit but the coefficient is not the expected -1. If we constrain the coefficient to be -1, the least squares estimate of the intercept is given as the average of $log(Latency_i log(s_i))$ **(green line, Figure S7).** We also consider forcing the intercept to take a more plausible value of $N^* = 100{,}000$ **(blue line Figure S7).**

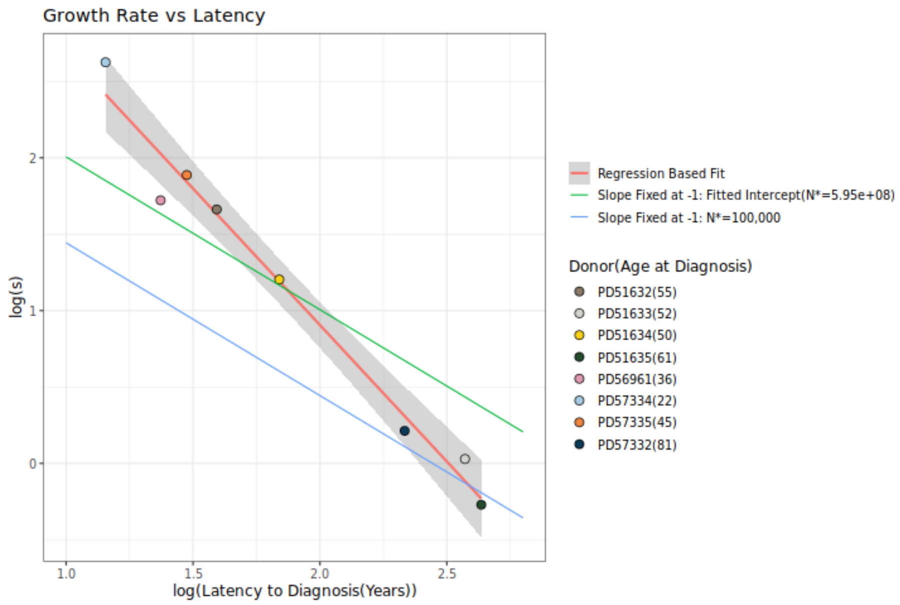

Figure S7 Net Growth rate vs Latency on a log-log scale. *The red line shows the model best fit line for log(s)~log(Latency) and the shaded grey area represents the 95% confidence intervals for the expected value of log(s). The green and blue lines represent the model fit if the slope is forced to be -1.*

One interpretation of the unexpected slope is that the effective growth rate between acquisition and diagnosis is lower than the fitted growth rate. Indeed, built into our *phylofit* growth rate estimation is an assumption that the mutant population size follows a logistic trajectory:

$$N(t) = \frac{N}{1 + e^{-s(t-t_m)}}$$

Whereas the above modelling assumes a pure exponential growth model:

$$N(t) = e^{rt}$$

Now, if we measure the growth rate between two points on the logistic growth curve we can estimate an effective exponential growth rate r that produces the same endpoint growth as the logistic curve between the two points. If we chose the two points, in a symmetric fashion around the midpoint, so that the starting population is a fraction ϵ (e.g. 1%) of the carrying capacity, and the second point is $1 - \epsilon$ (e.g. 99%) of the carrying capacity, then this implied rate of an exponential growth process is exactly half of the logistic growth s, so under this scenario we would expect that $log(r) = log(s) - log(2)$ and that the slope would remain unaffected **(Figure S8)** and so even a logistic growth trajectory, where the population saturates and growth slows down over time, does not account for the observed departure of the slope from -1.

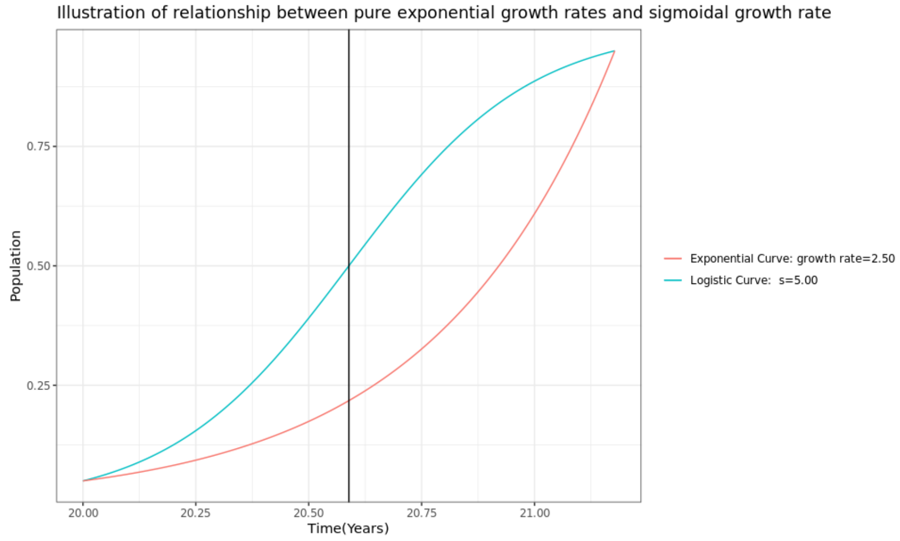

Figure S8 *A logistic and exponential curve when the population is 1% and 99% of the logistic curve saturation population and the exponential growth rate, r, is half that of the logistic growth rate, s.*

In summary, whilst there is a clear correlation between growth rate and latency to diagnosis in the cohort, our modelling suggests that patients do not simply present with disease when they reach a certain number of mutant HSCs following a period of clonal expansion that follows either exponential or logistic growth. Indeed, the latency to diagnosis observed in patients is more than expected under our simple model.

It is possible that some patients (e.g. younger patients) have increased carrying capacity of mutant HSCs, or increased tolerance of disease burden, before presenting with symptoms. Our observation could also be explained by there being a maximum number of leukemic HSCs that are able to be sustained in the bone marrow, thus more dramatically limiting population growth, particularly at extremely high s, as observed in some patients.

References

1. Mitchell, E. et al. Clonal dynamics of haematopoiesis across the human lifespan. Nature 606, 343–350 (2022).

2. Williams, N. et al. Life histories of myeloproliferative neoplasms inferred from phylogenies. Nature 602, 162–168 (2022).

3.Johnson, B., Shuai, Y., Schweinsberg, J. & Curtius, K. cloneRate: fast estimation of single-cell clonal dynamics using coalescent theory. Bioinformatics 39, btad561 (2023).

4. Mooers, A., Gascuel, O., Stadler, T., Li, H. & Steel, M. Branch Lengths on Birth–Death Trees and the Expected Loss of Phylogenetic Diversity. Syst. Biol. 61, 195–203 (2012).
