# Supplementary material for: Timing and trajectory of BCR::ABL1-driven chronic myeloid leukaemia
Source: Nature. 2025 Apr 9;640(8060):982–90. doi: 10.1038/s41586-025-08817-2 (PMC12018454; doi:10.1038/s41586-025-08817-2)
Supplement: Supplementary file 2 — Reporting Summary [file 41586_2025_8817_MOESM2_ESM.pdf]

nature portfolio

Corresponding author(s): Jyoti Nangalia

Last updated by author(s): 6 Feb 2025

Reporting Summary

Nature Portfolio wishes to improve the reproducibility of the work that we publish. This form provides structure for consistency and transparency in reporting. For further information on Nature Portfolio policies, see our Editorial Policies and the Editorial Policy Checklist.

Statistics

For all statistical analyses, confirm that the following items are present in the figure legend, table legend, main text, or Methods section.

n/a	Confirmed	
☐	☒	The exact sample size (*n*) for each experimental group/condition, given as a discrete number and unit of measurement
☐	☒	A statement on whether measurements were taken from distinct samples or whether the same sample was measured repeatedly
☐	☒	The statistical test(s) used AND whether they are one- or two-sided<br>*Only common tests should be described solely by name; describe more complex techniques in the Methods section.*
☐	☒	A description of all covariates tested
☐	☒	A description of any assumptions or corrections, such as tests of normality and adjustment for multiple comparisons
☐	☒	A full description of the statistical parameters including central tendency (e.g. means) or other basic estimates (e.g. regression coefficient) AND variation (e.g. standard deviation) or associated estimates of uncertainty (e.g. confidence intervals)
☐	☒	For null hypothesis testing, the test statistic (e.g. *F*, *t*, *r*) with confidence intervals, effect sizes, degrees of freedom and *P* value noted<br>*Give P values as exact values whenever suitable.*
☐	☒	For Bayesian analysis, information on the choice of priors and Markov chain Monte Carlo settings
☐	☒	For hierarchical and complex designs, identification of the appropriate level for tests and full reporting of outcomes
☒	☐	Estimates of effect sizes (e.g. Cohen's *d*, Pearson's *r*), indicating how they were calculated

Our web collection on statistics for biologists contains articles on many of the points above.

Software and code

Policy information about availability of computer code

Data collection	FASTQ files were generated by Novaseq sequencing machines.
Data analysis	Single-nucleotide substitutions (SNV) were called using the CaVEMan (Cancer Variants through Expectation Maximization) algorithm, version 1.15.2 (https://github.com/cancerit/CaVEMan). Filtering designed for quality control following processing through the Sanger low-input sequencing pipeline was also applied (https://github.com/MathijsSanders/SangerLCMFiltering). Small insertions and deletions were called using the Pindel algorithm as implemented in the cgpPindel workflow, version 3.10.0 (https://github.com/cancerit/cgpPindel). Copy number variants were called using the ASCAT algorithm as implemented in the ascatNgs workflow, version 4.5.0 (https://github.com/cancerit/ascatNgs). Brass and GRIDSS pipeline (https://github.com/cancerit/BRASS and Cameron at al. 2017) was used for calling structural variants. Per patient structural variant results were reviewed and visualised with gGnome (https://github.com/mskilab-org/gGnome) this allowed the reconstruction of derivative chromosomes in patients with complex events. To ascertain the consequence of translocations grass (https://github.com/cancerit/grass) was employed. To further investigate the consequence of exonic breakpoints we used SpliceAI (https://github.com/Illumina/SpliceAI), to predict the splicing probabilities for each respective fusion sequence. Reconstructed fusion and reference sequence was used as input in the "custom sequence" script (https://github.com/Illumina/SpliceAI), "raw" splice acceptor and donor probabilities from SpliceAI were converted to bedGraph format and reviewed on IGV v2.17.4 (https://igv.org/doc/desktop/) release 2.17.4. De novo mutation signature extraction was performed using HDP (https://github.com/nicolaroberts/hdp). The SBSblood signature50 was downloaded and collated with the PCAWG signatures (https://cog.sanger.ac.uk/cosmic-signatures-production/documents/COSMIC_v3.3.1_SBS_GRCh38.txt). Allele counts at SNV and Indel sites were carried out using vafCorrect (https://github.com/cancerit/vafCorrect). Mutations were mapped to phylogenetic branches using treemut (https://github.com/nangalialab/treemut). Temporal branch lengths and per driver mutation rates were inferred using rtreefit (https://github.com/nangalialab/rtreefit). The growth rate of BCR-ABL1 clones was estimated using the previously described PhyloFit approach (Williams et al. 2022). Telomerecat (version 4.0.2, https://github.com/cancerit/telomerecat) was used to estimate mean telomere length (bp). Unmatched somatic mutation identification and filtering: was

performed with CaVEMan in addition to standard filters, SNVs flagged with "VUM" (seen in panel of normals) were rescued, all SNV were required to have a CLPM=0 and ASMD >=140. Short insertions and deletions (indels) were called using cgpPindel with the standard WGS cgpPindel VCF filters applied, except the F010 Pindel filter was disabled as it excludes variants seen in panel of normals. Driver candidate variants were restricted to the 37 gene set described. To filter germline variants we retained only SNVs and indels with a gnomAD v3.1.2 (Chen et al. 2024) popmax allele frequency < 0.01 from (annotated using echtvar v0.2.0 Pedersen & Ridder 2023). The union of SNVs and indels was then taken and reads counted across all samples belonging to the individual using VAFCorrect. Bulk phylogeny reconstruction: we used DPClust (https://github.com/Wedge-lab/dpclust) algorithm to infer mutational clusters using SNVs (CaVEMan filtered for proximity to indels called by PINDEL) and copy-number/sample purity (Battenberg https://github.com/cancerit/cgpBattenberg) called for each sample using a matched normal sample. Ctree (https://github.com/caravagnalab/ctree) was used to perform an exhaustive tree search. Random effects meta-analysis: rma function in the "metafor" R package. Code for analyses can be found at https://github.com/nangalialab/CML.

For manuscripts utilizing custom algorithms or software that are central to the research but not yet described in published literature, software must be made available to editors and reviewers. We strongly encourage code deposition in a community repository (e.g. GitHub). See the Nature Portfolio guidelines for submitting code & software for further information.

Data

Policy information about availability of data

All manuscripts must include a data availability statement. This statement should provide the following information, where applicable:

- Accession codes, unique identifiers, or web links for publicly available datasets
- A description of any restrictions on data availability
- For clinical datasets or third party data, please ensure that the statement adheres to our policy

Genome assembly GrCH38 is available at https://www.ncbi.nlm.nih.gov/datasets/genome/GCF_000001405.26/. PCAWG signatures are available at https://cog.sanger.ac.uk/cosmic-signatures-production/documents/COSMIC_v3.3.l_SBS_GRCh38.txt. Sequencing files have been deposited in the European Genome-Phenome Archive (https://www.ebi.ac.uk/ega/home), under accession number EGAD00001015473 in line with Wellcome Sanger Institute data sharing, and all somatic mutation .vcf files will be uploaded to Mendeley doi: 10.17632/yg29vx2f35.1 for publication. Use of individual-level data in the All of Us program is available to researchers across the world through the Researcher Workbench, a cloud-based computing platform (https://www.researchallofus.org/register/). Summary-level data is available to the public through a data browser provided by the research program (https://databrowser.researchallofus.org/).

Research involving human participants, their data, or biological material

Policy information about studies with human participants or human data. See also policy information about sex, gender (identity/presentation), and sexual orientation and race, ethnicity and racism.

Reporting on sex and gender	The sex of all participants is detailed in the study.
Reporting on race, ethnicity, or other socially relevant groupings	No variables on race, ethnicity or other socially relevant groups
Population characteristics	Samples were obtained from patients with chronic phase of chronic myeloid leukaemia treated at Cambridge Universities NHS Trust. Patients were selected to include a wide range of ages at diagnosis and variable treatment outcomes and were aged 22 to 81 years of age. 6 males and 3 females were studied.
Recruitment	All participants were enrolled in the study "Causes of Clonal Disorders Study" following fully informed and written consent, and in line with the Declaration of Helsinki. Selection of patients was opportunistic based on hospital attendance and sample availability, and therefore, was biased towards attenders during sample collection, and those with samples already banked. The limitations and impact of sampling strategy is discussed in the paper.
Ethics oversight	The study was covered under NHS Research Ethics Committee approval 05/MRE/44 and 18/EE/0199.

Note that full information on the approval of the study protocol must also be provided in the manuscript.

Field-specific reporting

Please select the one below that is the best fit for your research. If you are not sure, read the appropriate sections before making your selection.

☒ Life sciences ☐ Behavioural & social sciences ☐ Ecological, evolutionary & environmental sciences

For a reference copy of the document with all sections, see nature.com/documents/nr-reporting-summary-flat.pdf

Life sciences study design

All studies must disclose on these points even when the disclosure is negative.

Sample size	No specific protocol was employed to establish the number of single-cell-derived colonies per patient prior to sample collection. We opted for more than 50 colonies per patient to ensure the inclusion of a significant number of both mutant and wild-type colonies for the calculation of

	mutation burden and tree-based HSC population growth parameters. 9 patients diagnosed with CML were chosen to represent different age groups at diagnosis and different treatment outcomes achieved.
Data exclusions	Some single-cell-derived colonies were excluded from the analysis for quality control reasons or because there was evidence indicating that they originated from the same cell (technical replicate).
Replication	We performed a validation of growth rates estimates of the BCR::ABL1-mutated clone using different parameters for Phylofit and the maximum likelihood approach from Johnson et al. 2023. Replication of sequencing data involved sequencing multiple colonies from the same individual as detailed in phylogenetic trees, to increase confidence in parameter estimation. No wet lab experiments were repeated.
Randomization	N/A- this is a descriptive study with no test versus control groups.
Blinding	No blinding was undertaken in this descriptive study. Interpretation was of high throughput unbiased data generation. No subjective assessment was involved that required blinding.

Reporting for specific materials, systems and methods

We require information from authors about some types of materials, experimental systems and methods used in many studies. Here, indicate whether each material, system or method listed is relevant to your study. If you are not sure if a list item applies to your research, read the appropriate section before selecting a response.

Materials & experimental systems

n/a	Involved in the study	
☒	☐	Antibodies
☒	☐	Eukaryotic cell lines
☒	☐	Palaeontology and archaeology
☒	☐	Animals and other organisms
☒	☐	Clinical data
☒	☐	Dual use research of concern
☒	☐	Plants

Methods

n/a	Involved in the study	
☒	☐	ChIP-seq
☒	☐	Flow cytometry
☒	☐	MRI-based neuroimaging

Plants

Seed stocks	N/A
Novel plant genotypes	N/A
Authentication	N/A
